# Late Campanian fossil of a legume fruit supports Mexico as a center of Fabaceae radiation

**DOI:** 10.1038/s42003-020-01533-9

**Published:** 2021-01-14

**Authors:** Naylet K. Centeno-González, Hugo I. Martínez-Cabrera, Héctor Porras-Múzquiz, Emilio Estrada-Ruiz

**Affiliations:** 1 Departamento de Zoología, Escuela Nacional de Ciencias Biológicas–Instituto Politécnico Nacional, Prolongación de Carpio y Plan de Ayala s/n, 11340, Ciudad de México, Mexico; 2 Museo Paleontológico de Múzquiz, Adolfo E. Romo 1701, La Cascada, 26343, Santa Rosa de Múzquiz, Coahuila Mexico

**Keywords:** Evolution, Palaeontology

## Abstract

Fabaceae is one of the most diverse angiosperm families and is distributed across the globe in a variety of environments. The earliest evidence of the family, previous to this work, was from Paleogene sediments where it was found to be diverse in many fossil assemblages around the world. Here, we describe a fossil legume fruit from the Olmos Formation (upper Campanian) in northern Mexico. We designated the fossil fruit as *Leguminocarpum olmensis* Centeno-González, Martínez-Cabrera, Porras-Múzquiz et Estrada-Ruiz sp. nov., and related it with the Fabaceae family based on the presence of a dehiscent pod with two valves, an apex bearing stylar base, short stipe, and reticulated veins in the pericarp. We propose a new fossil species of *Leguminocarpum* for this fossil fruit. This fossil provides critical information on the long geologic history of Leguminosae around the world, significantly extending the record into the Cretaceous of Mexico.

## Abstract (web summary)

Naylet Centeno-González et al. report the discovery of the oldest legume fruit fossil at 73.5 million years old in the Olmos Formation of Mexico. This finding supports the hypothesis that Mexico is the center of radiation for Fabaceae.

## Introduction

The legume family is the result of one of the most spectacular radiations of flowering plants. In terms of the number of species, Fabaceae is the third most diverse family, only behind Asteraceae and Orchidaceae, including ca. 730 genera and ca. 19,400 species^1,2^, and represents one of the most ecologically diverse groups^3^. The Fabaceae is widely distributed throughout the world, especially in biomes such as the tropical rainforests and the dry forests of America.

Until now, there were no fossils fruits unequivocally belonging to the Fabaceae before the Paleogene^4^. However, by the Paleocene, the family was already diverse in many fossil assemblages around the world^4–9^. In North America, legume fossils are known beginning around 65.35 mya^9^. These records include genera found in current warm-temperate forests in the southeastern United States, but also genera restricted to the present day tropics^5^. The Fabaceae fossil record in Mexico is extensive and includes a wealth of vegetative and reproductive organs^10–16^.

In this paper, we described a fossil fruit of a legume from the upper Campanian in northern Mexico. The fruit has a combination of characters that relates it to different Fabaceae subfamilies such as Cercidoideae, Detarioideae, Caesalpinioideae and Papilionoideae. The legume family has a rich fossil record around the world, especially in Eocene and younger sediments, but this fossil significantly extends the record into the Cretaceous of Mexico.

## Results and discussion

**Systematic description.**

Family—Fabaceae

Genus—*Leguminocarpum* Dotzler

Species—*Leguminocarpum olmensis* sp. nov. Centeno-González, Martínez-Cabrera, Porras-Múzquiz et Estrada-Ruiz

### Etymology

The specific epithet refers to the Olmos Formation, where the fossil was collected.

### Fossil material

**Fig. 1:**
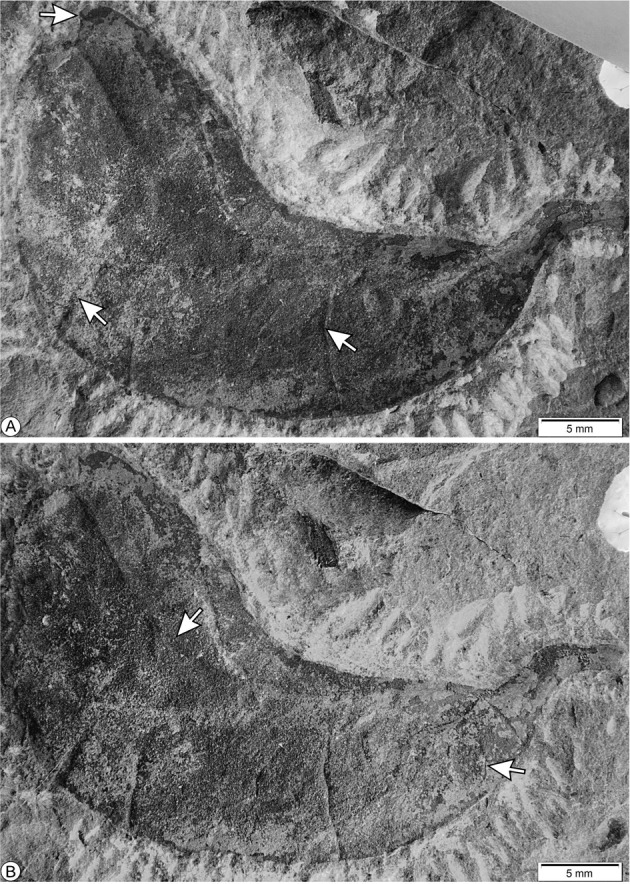
Fossil fruit from the Olmos Formation, Holotype MUZ-3907. **a** General view of the fossil specimen, showing stylar base (upper arrow), and fracture marks (bottom arrows). **b** A partially opened suture (upper arrow), and patterns of venation (bottom arrow).

**Fig. 2:**
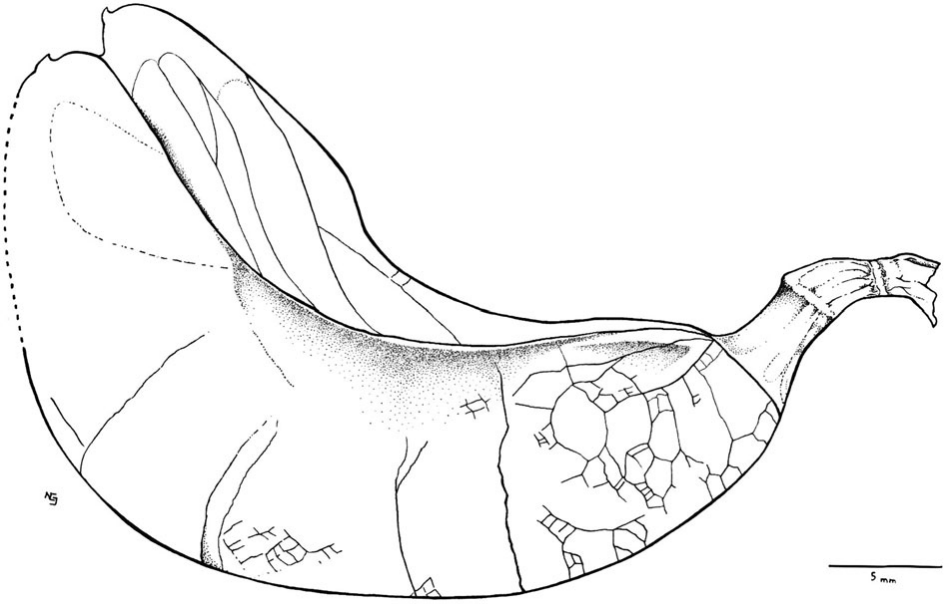
Line-drawing of Holotype (MUZ-3907). Reconstruction of the fossil fruit, showing fracture lines in the frontal valve, some veins in the epicarp, as well as the suture by which the valves are partially joined. Dotted lines represent areas where the fruit is partially visible.

**Fig. 3:**
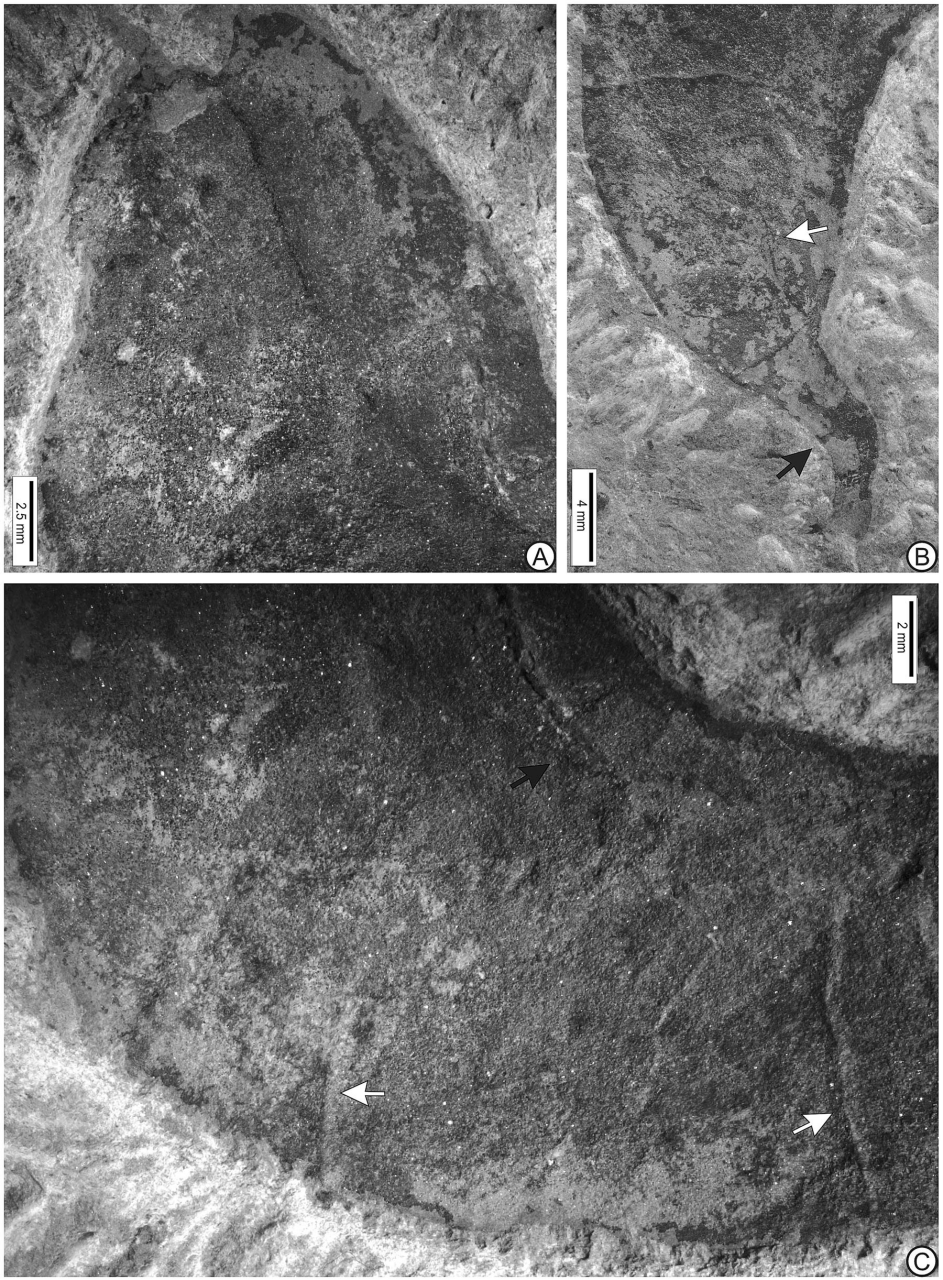
Detail of the fossil sample, Holotype MUZ-3907. **a** Showing of the apex of fruit. **b** Base of the fruit, preserving reticulated veins (white arrow), and the stipe (black arrow). **c** Suture (black arrow), and fracture marks (white arrows).

Holotype MUZ-3907 (Figs. 1–3).

### Locality

Olmos Formation (73.5 mya). Tajo La Florida, near the town of Melchor Múzquiz, Coahuila, with coordinates 27° 39′ 32.1″ N, -101° 19′ 10.7″ W.

### Stratigraphy

Upper Campanian, Olmos Formation.

### Place of deposit

Museo de Paleontología de Múzquiz, Melchor Múzquiz, Coahuila, Mexico.

### Species diagnosis

Legume fruit, asymmetric, curved shape; 3–4 times longer than wide; apex rounded, right angled, bearing a stylar base; base rounded to tapered, right-angled; short stipe; compressed; dehiscent along both sutures; without visible chambers, epicarp glabrous, reticulated veins, with ribs; wingless.

### Description

The description is based on one specimen (Figs. 1–3). The fossil preserves a partial oblique view and therefore it is possible to distinguish both valves joined by a suture. The fruit is strongly asymmetrical, with curved shape. Both sutures parallelly curved without twists; the partially opened suture is thick (Figs. 1–3). Margin not constricted. The fruit is 3 to 4 times longer than wide, the length of the fruit is 4.55 cm, and the maximum width of the valve is 1.25 cm; the smallest width near both the apex and base is 0.9 cm. The fruit is at least partially dehiscent. The two valves have separated in the apical part of the fruit and have shifted laterally, the style base appears on each valve (Figs. 1–3a). The apex is rounded and likely aligned to the partially opened suture, bearing a stylar base (Figs. 1a–3a). Base shape is rounded to tapered and right-angled. The fruit preserved the stipe with 4 mm long, both receptacle and pedicel total measure is 6.5 mm in length and 2.5 mm in diameter (Fig. 3b). The fruit is compressed, and does not have visible chambers. The fossil preserves numerous veins that arise from the sutures, forming reticulated veins closely spaced, oriented at an angle of approximately 90° relative to the dorsal and ventral sutures (Figs. 1b, 2, 3b, c). The valves have fracture marks, perpendicularly oriented to both sutures (Figs. 1a, 2, 3c).

### Taxonomic commentaries

The fossil fruit from the Olmos Formation was compared with Lardizabalaceae, Ranunculaceae, Apocynaceae, Proteaceae, Bignoniaceae, Annonaceae, and Fabaceae families. The fruits of Lardizabalaceae have fleshy follicles, dehiscent or indehiscent, and their shape elongate-oblong or subglobose. Examples of fruits with a possible morphological resemblance with the fossil in Lardizabalaceae were *Decaisnea* Hook. f. and Thomson and *Holboellia* Wall. *Decaisnea* has, however, features not present in the fossil such as woody epicarp, globose, and dehiscence along one suture. *Holboellia*, in addition, further differentiates from the fossil in having tuberculose epicarp, and a thick stipe. Despite these general similarities in shape and apex, the globose valves fruits, and the dehiscence along one suture prevents the inclusion of the fossil in the family.

Fruits in Ranunculaceae include achenes, berries, or follicles. Among the genera with some similarity with the fossil were *Helleborus* L., *Delphinium* L., *Aquilegia* L., and *Actea* L. Among the few similarities shared with the fossil, were the glabrous epicarp and the apex with a large beak. Because of the dehiscence along only one suture, differences in the general form, as well as the high prevalence of achenes in Ranunculaceae, *L. olmensis* bears no affinity with this family.

Fruits in Apocynaceae have some features in common with the fossil, specifically with *Gonolobus* Michx., *Cynanchum* L., *Matelea* Aubl., *Calotropis* R. Br., and *Tabernaemontana* L. *Gonolobus* has right-angled apex and base, large tapering, and conspicuously angled sutures. *Cynanchum* is smooth, lanceolate-ovoid in shape, 11–16 cm in length, thus setting it apart from the fossil. *Matelea* has fruits 9–11 cm in length and epicarp muricate. *Calotropis* has large fruits, 6–12 cm in length and 3–7 cm in width, rounded-ovate in shape, sub-globose and bladdery. *Tabernaemontana* has globose, straight-curve or rounded fruit, dehiscent along one suture. Although some sets of features present in Apocynaceae fruits resemble the fossil, they are different in size (up to 5 cm), the tubercular or or longitudinal ribs in the epicarp, and globose shape in transection, with curve-rounded shape, or curve with apex large with a beak, and thus we rule out any affinity.

Fruits in the Proteaceae family are fleshy, or non-fleshy, woody, with the carpel dehiscent along one suture, or indehiscent, there are follicles, drupes, or achenes. The follicles have similarities with the fossil only in the apex features, as well as in the presence of the stipe. However, the epicarp features, the globose valves (such as in *Telopea* R. Br., *Xylomelum* Smith and Sm., *Persoonia* Sm., *Hackea* Schrad. and J.C.Wendl., and *Grevillea* R. Br. ex Knight), and the general shape on fruit were distinct from the fruits from the Olmos Formation.

Bignoniaceae has fruits with two valves, but differs with the fossil in having linear or lanceolate or rounded shape, as well as the presence of a stronger longitudinal vein parallel to the valves, or a glabrous epicarp without veins. Many of these fruits with valves are linear or lanceolate in shape, being longest than wide, this is the case of *Bignonia* L., *Tabebuia* Gomes ex DC., *Pyrostegia* C. Presl, *Lundia* DC., *Dolichandra* Cham., *Fridericia* Mart., *Mansoa* DC., *Tanaecium* Sw., *Campsis* Lour., and *Handroanthus* Mattos. In some Bignoniaceae fruits the epicarp were pubescent or woody. Other fruits are distinct form than linear or lanceolate, such is the case of *Amphitecna* Miers, *Anemopaegma* Mart. ex Meisn., *Amphilophium* Kunth, *Jacaranda* Juss., *Pithecoctenium* Mart. ex Meisn., and *Kigelia* DC. *Amphitecna* has a curve-ovate shaped, non-stipate, rounded or short tapered-truncate base, globose, with 1 stronger longitudinal vein parallel to the valves, and glabrous in epicarp, without veins. *Anemopaegma* is tapered in base and apex, large beaked, stipate, and dehiscent, with a parallel line along the sutures. *Amphilophium* is subterete, with curve shape, two times longer than wide, short tapered base and substipate, ligneous epicarp without veins, and multiple seeds. *Jacaranda* is rounded in shape, compressed, bearing stylar base, rounded in base and apex, with longitudinal line parallel to valves, non-stipate. *Pithecoctenium* has a stylar base, nevertheless is a non-stipate fruit, with epicarp spinose, and globose transection. *Anemopaegma* is substipate fruit, short tapered in the base, and has a longitudinal line parallel to valves, the epicarp is coriaceous and glabrous. *Kigelia* is a curve, globose fruit, woody in epicarp, non-stipate, with rounded base and apex. Because of these differences we concluded the fossil is not related to this family.

Anonnaceae has some species with two valves and some genera bear stylar base. Nevertheless the fruits in this family are indehiscent, fleshy and globose shape in transection, the epicarp is glabrous, tuberculose, pubescent or spinose, without veins, being non-stipate or bearing a thick stipe. These characteristics are not present in the fossil fruit and are evident in genera such as *Xylopia* L., *Meiocarpidium* Engl. and Diels, *Orophea* Blume, *Monanthotaxis* Baill., *Uvaria* L., *Cymbopetalum* Benth., *Klarobelia* Chatrou, and *Uvariopsis* Engl.

**Fig. 4:**
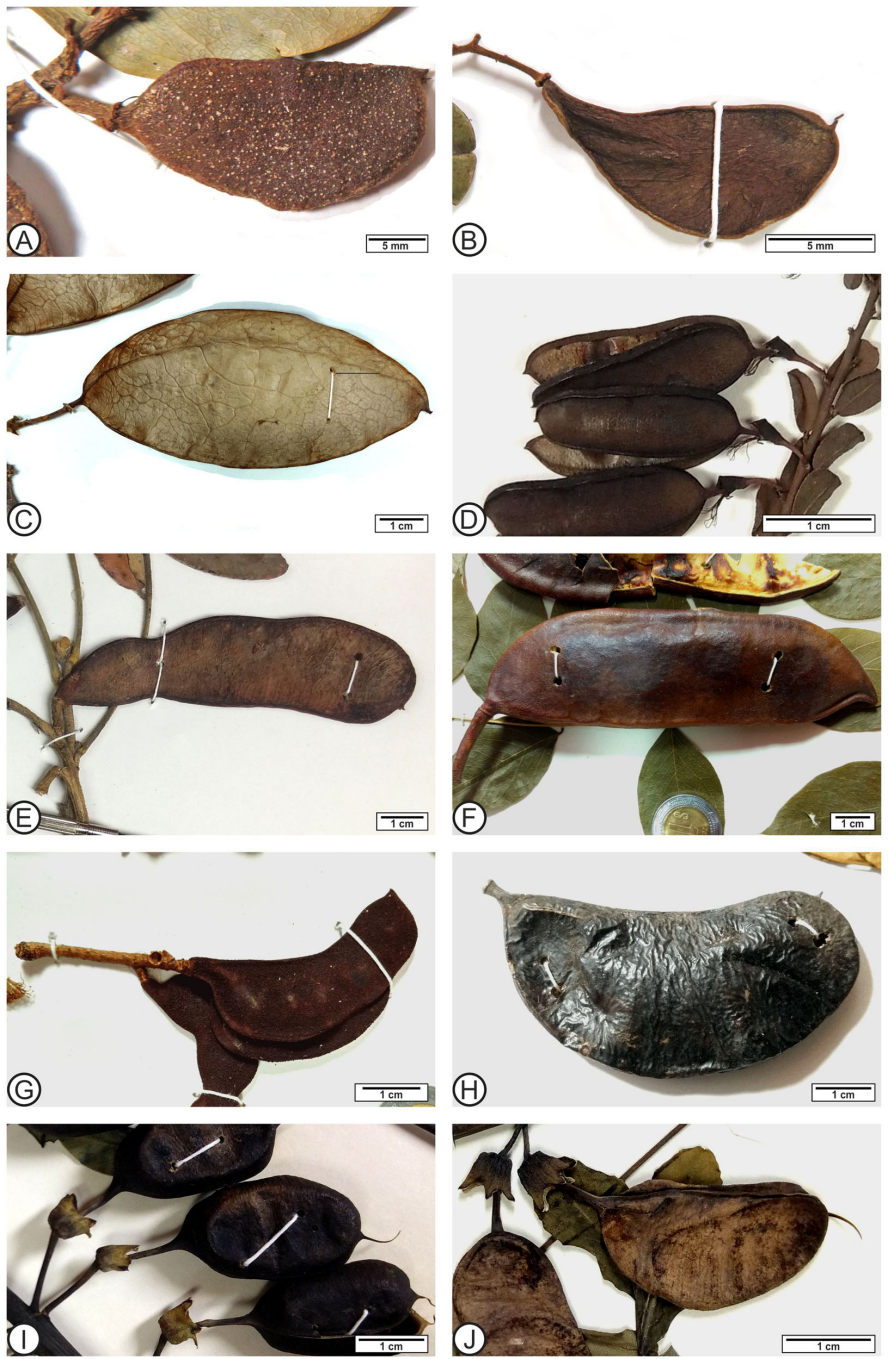
Extant samples of Fabaceae. **a** *Cynometra oaxacana* Brandegee (Detarioideae: 1509871–MEXU). **b** *Peltogyne mexicana* Martínez. (Detarioideae: 1032703–MEXU). **c** *Barnebydendron riedelii* (Tul.) J.H. Kirkbr. (Detarioideae: 1003269–MEXU). **d** *Bossiaea rhombifolia* Sieber ex DC. (Papilionidae: 469928–MEXU). **e** *Harpalyce arborescens* Gray (Papilionidae: 579909–MEXU). **f** *Gymnocladus dioicus* (L.) K. Koch. (Caesalpinioideae: 892167–MEXU). **g** *Gleditsia amorphoides* (Griseb.) Taub. (Caesalpinioideae: 650165–MEXU). **h** *Microlobius foetidus* (Jacq.) M. Sousa and G. (Caesalpinioideae: 1019245–MEXU). **i** *Baptisia bracteata* Elliot (Papilionidae: 962897–MEXU). **j** *Baptisia lactea* (Rafinesque) Thieret (Papilionidae: 142687–MEXU).

Lastly, the most notable characteristics present in fruits belonging to all legume subfamilies are a single superior carpel with one locule, marginal placentation, one to many ovules in two alternating rows on a single placenta, and dehiscent or indehiscent pods^2,17^ (Supplementary Data 1). The main features in common among the legume genera more similar to *Leguminocarpum olmensis* were the proportion, length, and width of the fruit, compressed transection, stylar base preserved, not visible chamber, the epicarp features, and the absence of a wing. Other features shared between the fossil and the reviewed genera were base form, apex form, the shape of the fruit, length of the stipe, and type of dehiscence. In particular, the fossil fruits have characteristics present in extant fruits of Cercidoideae, Detarioideae, Caesalpinioideae and Papilionoideae subfamilies (Fig. 4, Supplementary Data 1), from these subfamilies, some extant genera sharing more features with the fossil fruits are *Calpocalix* harms, *Macrosamanea* Britton, Rose ex Britton and Killip, *Microlobius foetidus* (Jacq.) Sousa and G., Griffonia Baill., *Colophospermum* Kirk ex Léonard, *Baphiopsis* Benth. ex Baker, *Baptisia* Vent., *Bossiaea* Vent., *Bowdichia* Kunth, *Dalbergia* L.f., *Haplormosia* Harms, *Harpalyce* Moç., and Sessé ex D. C., *Isotropis* Benth. Despite the resemblance between *L. olmensis* and extant Fabaceae, it could not be placed in any subfamily, because the fossil shows features morphologically similar to four extant subfamilies (see Supplementary Data 1).

Globally, legume fossil fruits have been described from Cenozoic sediments worldwide^4,6,8–12,18–25^. The fossil fruits Mezoneuron claibornensis Herendeen and Dilcher, *Mezoneuron flumen-viridensis* Herendeen and Dilcher, and *Mezoneuron spokanensis* (Knowlton) Herendeen and Dilcher, share with *L*. *olmensis* the presence of an apex bearing stylar base, a tapered base, and the presence of veins in the epicarp^21^. However, the flat membranous winged samaras, and the short, or absent stipe in these three fossil species preclude any relationship with the fossil fruits from the Olmos Formation. *Apuleia herendeenii* Calvillo-Canadell and Cevallos-Ferriz shares with *L. olmensis* the dehiscent valves and lack of visible chambers^23^. However, *L. olmensis* has major differences, namely the parallel curvature in both sutures, the curved shape, the rounded to aligned apex, rounded to the tapered base with the stipe, and the configuration of the veins on the valves. *Podocarpium podocarpum* (Braun) Herendeen (Supplementary Data 1) shows features in common to the fossil fruits such as a glabrous with obliquely reticulate venation, without visible chambers, as well as the length of the stipe, and the apex form; nevertheless *P*. *podocarpum* presents other different characteristics such as in the size fruit, symmetric form of the fruit, and the base form. Recently, several Paleocene fruits belonging to Detarioideae have been described^8^, among the specimens described, the fossil fruit morphotype 8 shows some characteristics shared with the fossil fruits from the Olmos Formation such asymmetry, lack of wings, stipe, and stylar base. The morphotype 8, however, has an acute tapered base, obtuse-acute apex and epicarp venation straight and oblique^8^ (Supplementary Data 1). Other fossil fruits have some features restricting the resemblance to *L. olmensis*, this is the case of *Mezoneuron claibornensis* Herendeen and Dilcher^19^, *Crudia Grahamiana* Herendeen and Dilcher^20^, *Eliasofructus claibornensis* Herendeen and Dilcher^18^, *Prosopis lazarii* Magallón-Puebla and Cevallos-Ferriz^13^, *Lysiloma mixtecana* Magallón-Puebla and Cevallos- Ferriz^13^, *Mimosa tepexana* Magallón-Puebla and Cevallos-Ferriz^13^, *Sophora sousae* Magallón-Puebla and Cevallos-Ferriz^13^, *Reinweberia omithopoides* Magallón-Puebla and Cevallos-Ferriz^13^ (Supplementary Data 1).

The fossil fruits from the Olmos Formation show differences to other previously described fossils and extant fruits (Supplementary Data 1), and therefore we propose a fossil species in Leguminosae named *Leguminocarpum olmensis* Centeno-González, Martínez-Cabrera, Porras-Múzquiz et Estrada-Ruiz.

### Biogeographic significance

Because of the high generic diversity of the group in tropical America and Africa-Madagascar, these regions have been suggested, in what it is known as the Gondwana hypothesis, as the place of origin and radiation of Fabaceae during the Upper Cretaceous, when these continents were in close proximity^1,4,26^. After that period, and throughout the Cenozoic, legumes were thought to have migrated to South America and North America, leaving behind the early divergent genera in Africa^26–28^. The Gondwanan hypothesis, however, is not supported by advances in the understanding of continental drift and the availability of more precise phylogenies for legumes^1,29^, as well as the reinterpretation of Eocene fossils^4,5^. This phylogenetic evidence also indicates that many South American records considered in the past as early divergent taxa correspond instead to recent offshoots from the northern hemisphere radiations, in contrast to the Gondwanan hypothesis^1^. The earliest records of Fabaceae fossils from different regions in the world are taxa previously established towards the Paleocene-Eocene boundary^4,6–9,30–32^. Their widespread distribution at that time suggests an earlier origin and diversification^4,22,29,33^. In recent studies, the majority of molecular phylogenies have proposed the origin of the crown group of the family between 59 and 70 (92.1) mya^29,34–38^. *Leguminocarpum olmensis*, with an age of ~73.5 Ma, gives support to the age proposed by molecular dating analyses previously cited, pointing to an earlier origin and diversification in the fossil record of Fabaceae, granted that *L. olmensis* is not a member of the stem group.

In North America, the Leguminosae were abundant and diverse during the Paleogene, suggesting North America as an important region for the evolutionary history of the family^4^. In Mexico, Leguminosae fossils have been recorded in Cenozoic sediments^9,11,39,40^. These fossil species recorded in Mexico have been related to both subtropical and tropical extant taxa currently growing in Central and South America^41–43^. Furthermore, in Mexico, the extant family is the second most diverse family just behind Asteraceae, with 1850 extant species belonging to 139 genera^44^. This record of *Leguminocarpum olmensis* in northern Mexico significantly extends the presence of Fabaceae into the Cretaceous of Mexico, suggesting low latitude North America as a place for the early evolution of Leguminosae.

## Conclusions

The fossil fruit from the Olmos Formation is described as a Cretaceous species of *Leguminocarpum* in  Fabaceae. The majority of the features present in the fossil can be found in all subfamilies of Fabaceae. However, *L. olmensis* most closely resembles species in Cercidoideae, Detarioideae, Caesalpinioideae and Papilionoideae subfamilies, and therefore its placement at this taxonomical level is uncertain. Although its within family affinities are unknown, this fossil extends the record of legumes into the Cretaceous of Mexico, suggesting low latitude North America, particularly northern Mexico, as a place for the early evolution of Leguminosae.

## Methods

### Geological setting

The paratropical rainforest of the Olmos Formation (upper Campanian), represents one of the most diverse fossil floras in the Americas^45–51^. In the Olmos Formation, there have been identified gymnosperms, such as Cupressaceae^52,53^, and aquatic ferns including *Salvinia*, *Dorfiella*† and *Marsilea*^49,54,55^. Among the numerous angiosperm leaves collected in the formation, there are taxa belonging to Arecaceae, Araceae, Moraceae, Betulaceae, Magnoliaceae, Lauraceae, Rhamnaceae, Menispermaceae, Nelumbonaceae, Caprifoliaceae, and Violaceae^46,47,49,56,57^. In addition, based on permineralized angiosperm woods and stem, there are fossil genera placed in Arecaceae, Malvaceae, Fagaceae, Anacardiaceae, Lauraceae, Cornaceae, Ericales, as well as *Metcalfeoxylon*^47,58,59^.

The Olmos Formation is one of the formations in the Navarro Group, which is located in the Sabinas Basin. It represents a fluvial-deltaic system, and based on the study of its lithofacies and fossils, has four depositional sub-environments: (1) lithofacies A, rich in coal, suggesting it corresponds to a swampy area with restricted circulation; (2) lithofacies B, composed of shale and sandstone that may represent floodplain environments and/or lagoons with open circulation; (3) lithofacies C, composed of fine to medium grained sandstone, with organic matter and parallel lamination and representing a fluvial environment, probably braided rivers, as suggested by the geometry of the sandbars and channel fills within the facies, and finally; (4) lithofacies D, composed of cross-stratified sandstones, interpreted as channel facies and levee deposited by a meandering river^49,51,57^. The fossil fruit described here was collected in the lithofacies B.

The age of the Olmos Formation has been dated as upper Campanian to lower Maastrichtian, starting with planktonic foraminifera assemblages Rosita fornicata/stuatiformis^60^, and palynology studies^61^ suggesting Lower Maastrichtian. Some ammonites in situ indicated an Upper Campanian age^62^. Furthermore, low adjacent San Miguel Formation is considered as Upper Campanian based on indicator fossils^63,64^, meanwhile, upper adjacent Escondido Formation contains the bivalves *Exogyra costata* Say and *Pycnodonte mutabilis* (Morton), giving it the age of Lower Maastrichtian (Personal communication, Vega-Vera, 2006). The age of the formation has been based on detrital zircons collected in sandstones in the lithofacies B of the Olmos Formation, where the fruits were collected, yielded and age of ~73.5 Ma, Upper Campanian (Personal communication, Callejas-Moreno, 2019).

### Collection and processing of fossil material

The fossil fruit was collected in 2016 from the locality known as Tajo La Florida, with coordinates 27° 39′ 32.1” N, and 101° 19′ 10.7” W. This locality is a private open mine located northwest of the town of Múzquiz, Melchor Múzquiz municipality, Coahuila, Mexico.

The observations of the morphological characteristics of the fossil fruits, as well as obtain the deep of fruit embedded in the rock, it was taken photographs by slices of the fossil, helped by the Microscope Zeiss AXIO Zoom.V16, and photographed with both AxioCam MRc5 camera and SC100 digital camera 5 Mpix., and the program Zen 2012, blue edition. It was carried out a tomography with CT Scanner Phillips Brilliance, 64-slice, to see some internal structure, nevertheless, none internal structure was observed due to the composition and hardness of the material.

The morphological description was made following terminology provided by specialized literature^17,65–70^. We compared the morphology of the fossil fruit with different families with material from the Herbarium at the Escuela Nacional de Ciencias Biológicas, IPN, and National Herbarium (MEXU), Instituto de Biología, UNAM, and digital herbaria of Missouri Botanical Garden^71^, and Royal Botanical Garden^72^. Once obtained a better morphological resemblance between the fossil and the extant specimens from one particular family, we carried a detailed search consulting herbaria data from the Herbarium at the Escuela Nacional de Ciencias Biológicas, IPN, and National Herbarium (MEXU), Instituto de Biología, UNAM, Mexico, specialized literature^66,67,69,70,73,74^, as well as fossil records from different ages^8,13,18–25,69,75–78^. The species with closer similarity to the fossil fruit may are in Supplementary Data 1. Characteristics observed embraced the size of the fruit, general shape, apex, and the base shape, presence, and length of the stipe, transection, dehiscence of valves, visible or not visible chambers, as well as characteristics of pericarp and presence or absence wing. The fossil fruit described in this paper is deposited in the Museo de Paleontología de Múzquiz (MUZ-3907), Melchor Múzquiz, Coahuila, Mexico. This paleontological collection is formally certified by the Instituto Nacional de Antropología e Historia (INAH), which formally protects the Mexican paleontological patrimony.

### Reporting summary

Further information on research design is available in the Nature Research Reporting Summary linked to this article.

## Supplementary information

Description of Supplementary Files

Suplemmentary Data 1

Reporting Summary

## Data Availability

The authors declare that the data supporting the findings of this study are available in the paper and its Supplementary Data files. The specimen is held in Museo de Paleontología de Múzquiz, México with a specimen number MUZ-3907.
