# Supplementary material for: Late Campanian fossil of a legume fruit supports Mexico as a center of Fabaceae radiation
Source: Commun Biol. 2021 Jan 14;4:41. doi: 10.1038/s42003-020-01533-9 (PMC7809014; doi:10.1038/s42003-020-01533-9)
Supplement: Supplementary file 1 — Description of Supplementary Files [file 42003_2020_1533_MOESM1_ESM.pdf]

Description of Additional Supplementary Files

File name: Supplementary data 1

Description: Comparison table among extinct and extant Fabaceae genera with characters resembling Leguminocarpum olmensis. These characters could be present in different species of the showed genera. Information about character states was obtained from different authors, and by comparison of herbarium material (8, 13, 18-21, 23, 25, 66, 67, 69, 71-78). Observations about revised specimens from National Herbarium (MEXU), Instituto de Biología, UNAM, Mexico were included.
