# Supplementary material for: Late Campanian fossil of a legume fruit supports Mexico as a center of Fabaceae radiation
Source: Commun Biol. 2021 Jan 14;4:41. doi: 10.1038/s42003-020-01533-9 (PMC7809014; doi:10.1038/s42003-020-01533-9)
Supplement: Supplementary file 3 — Reporting Summary [file 42003_2020_1533_MOESM3_ESM.pdf]

nature research

Corresponding author(s): EER, COMMSBIO-19-1807

Last updated by author(s): Oct 26, 2020

Reporting Summary

Nature Research wishes to improve the reproducibility of the work that we publish. This form provides structure for consistency and transparency in reporting. For further information on Nature Research policies, see our Editorial Policies and the Editorial Policy Checklist.

Statistics

For all statistical analyses, confirm that the following items are present in the figure legend, table legend, main text, or Methods section.

n/a	Confirmed	
☒	☐	The exact sample size (*n*) for each experimental group/condition, given as a discrete number and unit of measurement
☒	☐	A statement on whether measurements were taken from distinct samples or whether the same sample was measured repeatedly
☒	☐	The statistical test(s) used AND whether they are one- or two-sided<br>*Only common tests should be described solely by name; describe more complex techniques in the Methods section.*
☒	☐	A description of all covariates tested
☒	☐	A description of any assumptions or corrections, such as tests of normality and adjustment for multiple comparisons
☒	☐	A full description of the statistical parameters including central tendency (e.g. means) or other basic estimates (e.g. regression coefficient) AND variation (e.g. standard deviation) or associated estimates of uncertainty (e.g. confidence intervals)
☒	☐	For null hypothesis testing, the test statistic (e.g. *F*, *t*, *r*) with confidence intervals, effect sizes, degrees of freedom and *P* value noted<br>*Give P values as exact values whenever suitable.*
☒	☐	For Bayesian analysis, information on the choice of priors and Markov chain Monte Carlo settings
☒	☐	For hierarchical and complex designs, identification of the appropriate level for tests and full reporting of outcomes
☒	☐	Estimates of effect sizes (e.g. Cohen's *d*, Pearson's *r*), indicating how they were calculated

Our web collection on statistics for biologists contains articles on many of the points above.

Software and code

Policy information about availability of computer code

Data collection	No computer code was used in this paper
Data analysis	No computer code was used in this paper

For manuscripts utilizing custom algorithms or software that are central to the research but not yet described in published literature, software must be made available to editors and reviewers. We strongly encourage code deposition in a community repository (e.g. GitHub). See the Nature Research guidelines for submitting code & software for further information.

Data

Policy information about availability of data

All manuscripts must include a data availability statement. This statement should provide the following information, where applicable:

- Accession codes, unique identifiers, or web links for publicly available datasets
- A list of figures that have associated raw data
- A description of any restrictions on data availability

The authors declare that the data supporting the findings of this study are available in the paper and its Supplementary data files. The specimen is held in Museo de Paleontología de Múzquiz, Coahuila, México with a specimen number MUZ-3907.

Field-specific reporting

Ecological, evolutionary & environmental sciences study design

All studies must disclose on these points even when the disclosure is negative.

Study description	The fossil fruit was collected in 2016 from the locality known as Tajo La Florida, with coordinates 27º 39' 32.1" N, and 101º 19' 10.7" W. This locality is a private open mine located northwest of the town of Múzquiz, Melchor Múzquiz municipality, Coahuila, Mexico.
Research sample	The fossil fruit described was collected in sediments of the Olmos Formation, in the lithofacies B, which is composed of shale and sandstone that may represent floodplain environments and/or lagoons with open circulation.
Sampling strategy	N/A
Data collection	The fossil fruit described in this paper is deposited in the Museo de Paleontología de Múzquiz (MUZ-3907), Melchor Múzquiz, Coahuila, Mexico.
Timing and spatial scale	N/A
Data exclusions	N/A
Reproducibility	N/A
Randomization	N/A
Blinding	N/A

Did the study involve field work? [x] Yes [ ] No

Field work, collection and transport

Field conditions	N/A
Location	Coahuila State, Mexico
Access & import/export	The fossil fruit described in this paper is deposited in the Museo de Paleontología de Múzquiz (MUZ-3907), Melchor Múzquiz, Coahuila, Mexico.
Disturbance	No, the fossil was collected in an open mine

Reporting for specific materials, systems and methods

We require information from authors about some types of materials, experimental systems and methods used in many studies. Here, indicate whether each material, system or method listed is relevant to your study. If you are not sure if a list item applies to your research, read the appropriate section before selecting a response.

Materials & experimental systems

n/a	Involved in the study	
[x]	[ ]	Antibodies
[x]	[ ]	Eukaryotic cell lines
[x]	[ ]	Palaeontology and archaeology
[x]	[ ]	Animals and other organisms
[x]	[ ]	Human research participants
[x]	[ ]	Clinical data
[x]	[ ]	Dual use research of concern

Methods

n/a	Involved in the study	
[x]	[ ]	ChIP-seq
[x]	[ ]	Flow cytometry
[x]	[ ]	MRI-based neuroimaging
